# Evolution After Whole-Genome Duplication: A Network Perspective

**DOI:** 10.1534/g3.113.008458

**Published:** 2013-11-01

**Authors:** Yun Zhu, Zhenguo Lin, Luay Nakhleh

**Affiliations:** * Department of Computer Science, Rice University, Houston, Texas 77005; † Department of Ecology and Evolutionary Biology, Rice University, Houston, Texas 77005

**Keywords:** whole-genome duplication, protein networks, yeast, duplication rate

## Abstract

Gene duplication plays an important role in the evolution of genomes and interactomes. Elucidating how evolution after gene duplication interplays at the sequence and network level is of great interest. In this work, we analyze a data set of gene pairs that arose through whole-genome duplication (WGD) in yeast. All these pairs have the same duplication time, making them ideal for evolutionary investigation. We investigated the interplay between evolution after WGD at the sequence and network levels and correlated these two levels of divergence with gene expression and fitness data. We find that molecular interactions involving WGD genes evolve at rates that are three orders of magnitude slower than the rates of evolution of the corresponding sequences. Furthermore, we find that divergence of WGD pairs correlates strongly with gene expression and fitness data. Because of the role of gene duplication in determining redundancy in biological systems and particularly at the network level, we investigated the role of interaction networks in elucidating the evolutionary fate of duplicated genes. We find that gene neighborhoods in interaction networks provide a mechanism for inferring these fates, and we developed an algorithm for achieving this task. Further epistasis analysis of WGD pairs categorized by their inferred evolutionary fates demonstrated the utility of these techniques. Finally, we find that WGD pairs and other pairs of paralogous genes of small-scale duplication origin share similar properties, giving good support for generalizing our results from WGD pairs to evolution after gene duplication in general.

Gene duplication is a major evolutionary event both for the genome sequence and for the protein−protein interaction (PPI) network growth. It is considered to be a major contributor to shaping and refactoring the functionalities of the organism and thus has been widely studied especially in terms of its role in evolution. After the seminal work of (Ohno 1970), more and more analyses have been conducted and more models have been developed for gene duplication on the basis of ever-increasing data sources (Dittmar and Liberles 2010). Among all the studies, some focused on gene duplication from sequence level, and to estimate, for example, probabilities, timings, and rates of duplication events (Pál *et al.* 2005; Pinney *et al.* 2007; Paps *et al.* 2009). Some focused at the role of duplication in network evolution and proposed graph-theoretic models of network growth, such as the duplication-attachment model (Wiuf *et al.* 2006) and duplication-divergence model (Bhan *et al.* 2002; Zhang *et al.* 2006; Ratmann *et al.* 2007). Several other studies also have explored how duplicated genes maintain, lose, or modify their functions (Jukes and Cantor1969; Gibson and Goldberg 2008; Innan and Kondrashov 2010; Li *et al.* 2012).

From a network (*e.g.*, protein−protein interaction, or PPI, network) perspective, gene duplication results in the birth of new gene copy whose connections in the network are identical to those of the ancestral copy immediately before duplication. After gene duplication, because of the accumulation of different mutations on each of the duplicated pair, gain and loss of PPI connections in the network would be expected. However, little is known about how mutations at the sequence level of a duplicate gene pair would affect the evolution of an interaction network. Qian *et al.* (2011) experimentally examined 87 potential interactions between *Kluyveromyces waltii* proteins, whose one-to-one orthologs in the related budding yeast *Saccharomyces cerevisiae* have been reported to interact. In their study, duplicated genes are avoided to obtain the one-to-one correspondence in two different species. In other words, while this study considered network evolution and its rate, it focused on orthologs and deliberately excluded paralogs.

Given the central role that duplication plays in the evolution of interaction networks, it would be interesting to understand how networks shed light on the evolution of gene duplicates, and how to estimate evolutionary rates of network evolution by using duplicated genes. To investigate these issues, we focus on the whole-genome duplication (WGD) in yeast. An ancestor of *S. cerevisiae* underwent a WGD event (Wolfe and Shields 1997; Kellis *et al.* 2004). Only approximately 10% of WGD gene pairs (550 pairs) are still present in the extant *S. cerevisiae* genome (Kellis *et al.* 2004). Because the duplication of these survived WGD gene pairs occurred at the same time and their sequence evolved at potentially different rates, these WGD gene pairs can be used as ideal subjects to learn how the evolution rate varies among different gene duplication pairs at both sequence level and network level.

Here, we investigated the evolutionary rates of the different WGD pairs and found some variations in these rates, although within a small range. Correlating these rates with sequence, network, and fitness data, we found that gene expression and fitness correlate strongly with evolutionary rates of WGD duplicates. As essentiality and redundancy of genes interplay with expression and fitness effects, we set out to understand this interplay using WGD pairs. We first established rates of gain/loss of network interactions by using sequence divergence. We also developed a model of correlation between sequence divergence and network divergence, which captures the synchronized evolution at the sequence and network levels. Then, we used network local topologies (neighborhoods of WGD pairs) as proxies for functional similarity and divergence. On the basis of this connection between local topologies and functional similarities, we developed an expectation-maximization algorithm and learned the evolutionary fates of WGD pairs and correlated them with epistatic effects. Our results reveal the extent of conserved functionalization (CF), subfunctionalization (SF), and neofunctionalization (NF) that ensued after WGD. Furthermore, epistatis analyses correlated well with the inferences made.

Our results demonstrate the power of WGD as “calibrated” data points to investigate network evolution and the use of networks and their topologies to shed light on evolution after gene duplication, and in particular, after WGD. We find gene pairs that arose due to WGD have similar properties to those of gene pairs that arose due to small-scale gene duplication events. This observation further generalizes our results from evolution after WGD to evolution after duplication.

## Methods

### An EM algorithm for determining the fate of WGD gene pairs

From a network perspective, the fate of a WGD gene pair can be inferred from the shared neighborhoods of the pair. To achieve this task, we developed an expectation-maximization (EM) that is inspired by the work of Zeng and Hannenhalli (2013). The original method of Zeng and Hannenhalli (2013) characterizes function by tissue-specific gene expression level, whereas we characterize function by normalized neighborhood sizes. The approach of Zeng and Hannenhalli (2013) does not work here because they use sequence similarity of paralogs to construct a phylogenetic tree whose branch lengths serve as a surrogate of time since duplication. We, instead, target WGD pairs in which all the genes were duplicated at the same time.

Our EM algorithm works as follows. Let the neighborhood of paralog genes *g*_1_ and *g*_2_ be both *N*_0_ right after duplication, and be *N*(*g*_1_) and *N*(*g*_2_) be the neighborhoods at present. Let the size for normalization be *ttl* = |*N*(*g*_1_) ∪ *N*(*g*_2_)| and define

$$x=\frac{\left|N\left(g_{0}\right)\right|}{ttl}\;a=\frac{\left|N\left(g_{1}\right)\right|}{ttl}\;b=\frac{\left|N\left(g_{2}\right)\right|}{ttl}\;sh=\frac{\left|N\left(g_{1}\right)\cap N\left(g_{2}\right)\right|}{ttl}.$$

Under pure CF, we expect *a* = *b* = *x* = *sh* = 1; under pure SF, we expect *a* + *b* = x = 1, *sh* = 0; and, under pure NF, we expect *a* = *x* (or *b* = *x*) and *a* + *b* = 1 > *x*, *sh* = 0. We further normalize the three values by their maximum value as follows:

$$x=\frac{x}{\text{max}\left(x,a,b\right)},\;a=\frac{a}{\text{max}\left(x,a,b\right)},\;b=\frac{b}{\text{max}\left(x,a,b\right)}.$$

The probabilistic model for classification can be captured as: (1) SF: *a* + *b* = 1; (2) CF: *a* + 1 = 2*x*; and, (3) NF: *x* = *a*, *x* ≤ 0.5. For any given *x*, *y*, and *z* values, it is classified by a plane for the feature points.

Suppose that under CF model, rate of losing one of the two edges originated from duplication is *μ_d_*, and rate of gaining a new edge is *μ_a_*; under SF model, rate of losing one of the two edges originated from duplication is *μ_D_*, and rate of gaining a new edge is *μ_a_*; under NF model, rate of losing one of the two edges originated from duplication is *μ_D_*, and rate of gaining a new edge is *μ_A_* (assuming neofunctionalizaiton is accompanied by subfunctionalization [SF]). In general, *μ_D_* > *μ_d_* and *μ_A_* > *μ_a_*.

Let *θ* be the set of parameters, including all the *μ* values listed previously. Let *Z*(*g*_1_, *g*_2_) ∈ {*CF*, *SF*, *NF*} be the fate for WGD gene pair *g*_1_ and *g*_2_, and *sh*(*g*_1_, *g*_2_) be the observed normalized shared neighborhood size (|*N*(*g*_1_) ∩ *N*(*g*_2_)|/*ttl*). We then apply the standard EM framework as follows:

1. Initialize the parameters *θ* to some random values.
2. Compute the best value for *Z* given these parameter values. That is, according to the probabilistic model for classification, under current *θ* value, infer the most probable fate for each WGD gene pair.
3. Use the computed values of *Z* to compute a better estimate for the parameters *θ*.
4. Repeat steps 2 and 3 until converge.

To avoid local maxima, we repeated the process with several different starting values of *θ*.

## Results

All results reported herein are based on WGD pairs of genes and PPI) data from *S. cerevisiae*. The PPI data were downloaded from the DIP database (Xenarios *et al.* 2000), which has high confidence value for links (interactions). To validate our results, we also used the low-throughput links and links supported by more than a single high-throughput experiment in the BIOGrid database (Stark *et al.* 2006). The sequence and gene family data were downloaded from Butler *et al.* (2009).

### Sequence divergence of WGD pairs

As we set out to use a set of whole-genome duplication pairs, or WGD pairs for short, we first inspected the variability across WGD pairs in terms of sequence divergence, mutation rates, and other properties. Consider two sequences that have diverged for time *t*, and let *r* be the mutation rate per site. Further, assume that the observed normalized distance between the two sequences is *p* (that is, *p* is the proportion of sites at which the two sequences differ). Assuming equality of substitution rates among sites and equal amino acid frequencies, we have the relationship (Nei and Kumar 2000)

$$\left(1-p\right)=e^{-2rt}.$$

For *S. cerevisiae* WGD pairs, *t* is estimated to be approximately 100 million years (Wolfe and Shields 1997). Given that we can compute *p* from the WGD pairs, we can compute the mutation rate *r* for each pair of WGD gene sequences as

r=−ln(1−p)/(2∗t).

Because *t* is the same for all WGD gene pairs, in this work we will compute *rt* instead:

rt=−ln(1−p)/2. (1)

**Figure 1:**
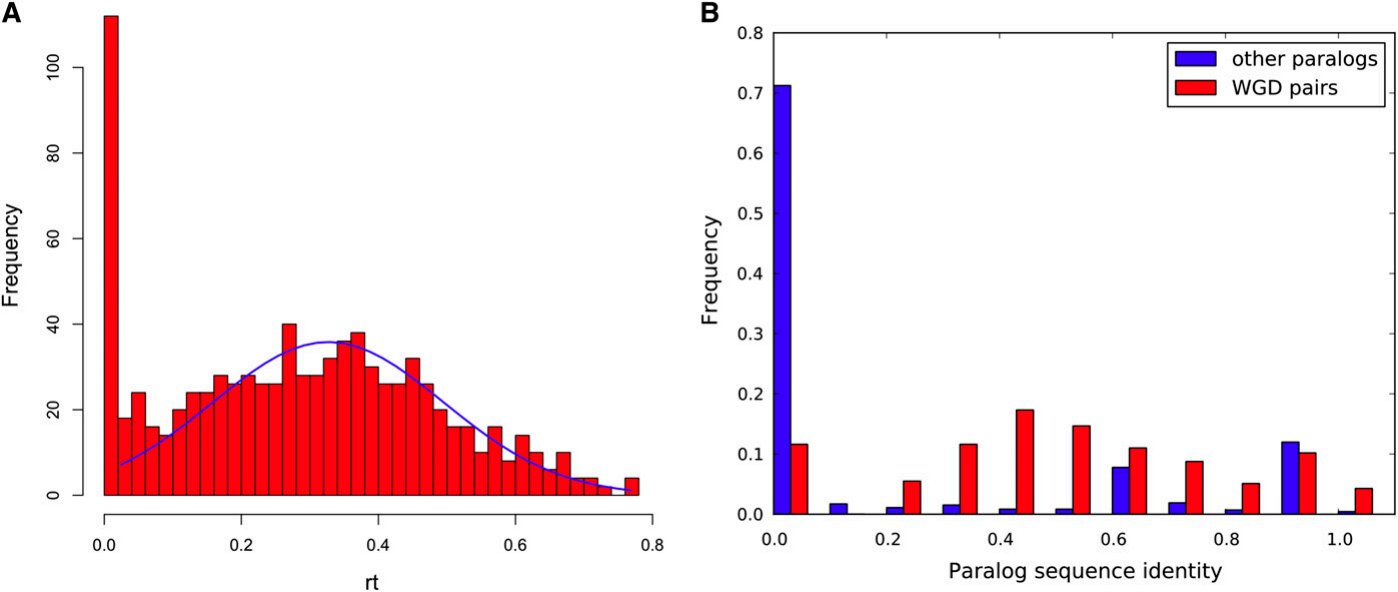
(A) Distribution of *rt* of WGD pairs with normal curve fitting (mean = 0.3268, SD = 0.1685). (B) Distribution of proportion of sequence identity for WGD pairs and pairs of other paralogs. Because the duplication time of “other paralogs” pairs is unknown, we do not use *rt* here.

The distribution of *rt* values of WGD pairs is given in Figure 1.

As Figure 1 shows, a normal distribution with mean 0.3268 and SD as 0.1685 gives a good fit for the data. Notice that a big portion of WGD pairs have *rt* values that are close to 0, which means a large portion of WGD pairs do not diverge much from each other. Also notice that the overall possible value for *rt* is within a relatively small range ([0, 0.8]), which means the mutation rate for different WGD pairs are not very different from each other.

From Figure 1A, we can see that the *rt* values of WGD pairs can be fitted to a normal curve except for the peak at *rt* = 0. Because *rt* here is computed based on the equation 1 − *P* = *e*^2^*^rt^* and 1 − *p* is the sequence identity, we plotted the distribution of sequence identity proportions in Figure 1B for both WGD pairs and other paralogous pairs (pairs of paralogs that are the result of a small-scale duplication event). Although non-WGD paralogous pairs have different times of duplication, the overall trend shows that WGD pairs have much greater paralog sequence identity, which could mean that either the mutation rate *r* is smaller for WGD pairs than for non-WGD pairs, or that many of the individual small-scale duplication events are more recent than the WGD event.

One caveat of observing high level of sequence identity for WGD pairs is that WGD pairs may have gone through a significant amount of interlocus gene conversions. At least 10% of WGD pairs in yeast have experienced gene conversion and the average time length of concerted evolution is about 58~75 million years (Z. Lin, unpublished data), which could potentially result in a small shift to the right in Figure 1A. It is important to note that different non-WGD paralogous pairs originated from duplication events at different times.

### What influences the evolutionary rates of different WGD pairs?

**Figure 2:**
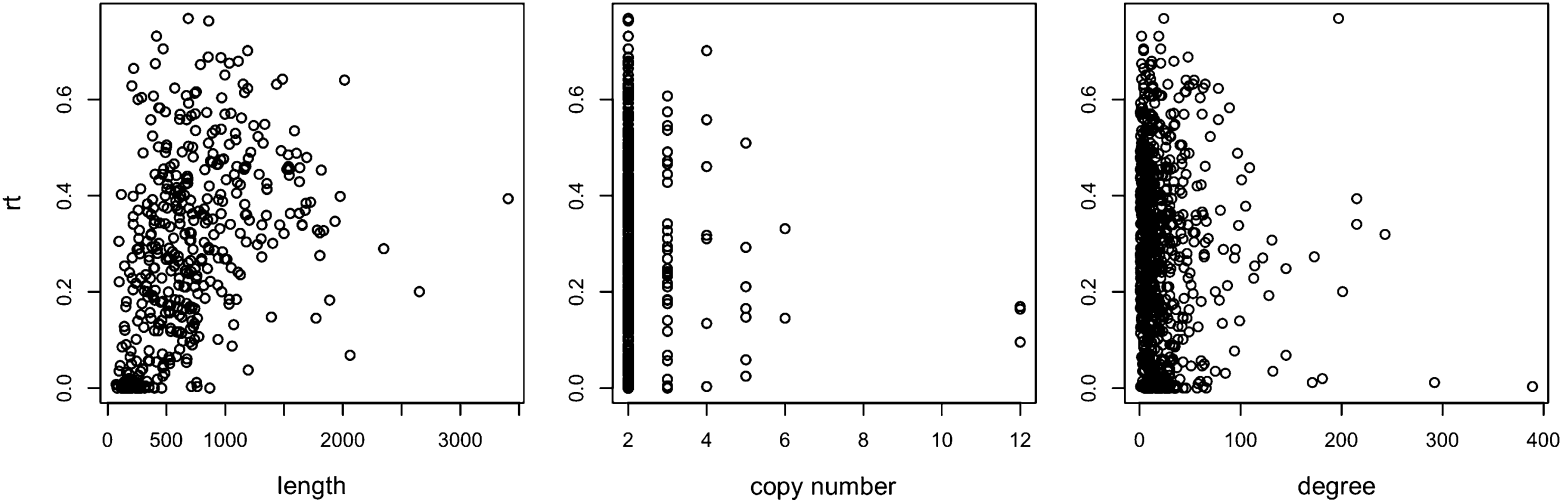
For the set of WGD pairs, the lengths of gene sequences, the number of copies within the families, and the degree of the genes, respectively, are shown against *rt*.

As we stated previously, it seems that the mutation rates are not very different for the different WGD pairs. Still, variability exists in the rates, and the question is: what factors play a role in this variability? To answer this question, we correlated the divergence rates of WGD pairs with three metrics: the length of gene sequences, the number of gene copies in the family, and the degree of the gene in the PPI network. The results are shown in Figure 2.

We calculated Pearson’s correlations for data in each of the three panels. The correlation between *rt* and the gene sequence length is 0.261, with *P* = 0.0002, which implies that WGD pairs of longer gene sequences diverge more than pairs with shorter gene sequences. This finding makes sense because *r* is the mutation rate per site, and longer gene sequences accumulate more mutations and result in greater degrees of divergence between the genes involved in a WGD pair. The correlation between *rt* and the copy number is −0.071, with *P* = 0.1382, which indicates almost no correlation between the two. The correlation between *rt* and the average degree of WGD pairs is −0.135, with *P* = 0.0005, which implies that WGD pairs with greater connectivity diverge slower at the sequence level. However, this might be a case of cause-effect: certain WGD pairs evolve slower, resulting in the loss of fewer neighbors, and thus greater connectivity. Furthermore, this negative correlation between divergence and connectivity is reasonable because an increase number of mutations, particularly those in regions involved in the interactions, would result in an increased (albeit not necessarily at the same rate) loss of interactions. This agrees with recent findings on how mutation at the genomic level, combined with neutral evolutionary forces, shape emergent properties at the network level (Ruths and Nakhleh 2013) and can explain correlations between network properties and gene duplicability (Zhu *et al.* 2012).

**Figure 3:**
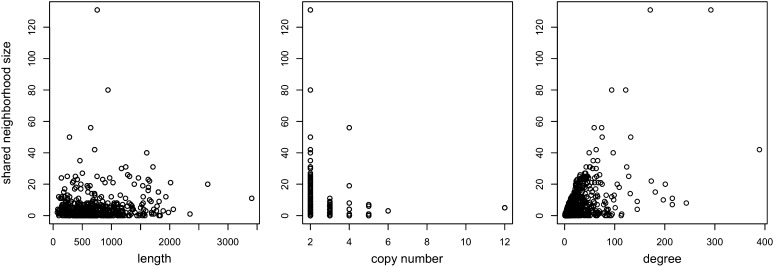
For the set of WGD pairs, the lengths of gene sequences, the number of copies within the families, and the degree of the genes, respectively, are shown against *sh*(*g*_1_, *g*_2_).

Furthermore, we used the shared neighborhood size as a measure of gene divergence at the network level and conducted a series of similar analyses to understand whether there is a correlation between “network-level divergence” and those properties. For a given gene *g*, we denote by *N_t_*(*g*) the set of all neighbors of gene *g* in some protein interaction network of interest at time *t* during evolution. Consider two paralogous genes, *g*_1_ and *g*_2_, where *g*_2_ is duplicated from *g*_1_ at time 0. We denote by *sh_t_*(*g*_1_, *g*_2_) = |*N_t_*(*g*_1_) ∩ *N_t_*(*g*_2_)| denote the size of the shared neighborhoods of *g*_1_ and *g*_2_ at time *t*. In this part, because we are considering pairs of extant WGD pairs, we drop the *t* in the subscript. Figure 3 shows the gene length, copy number, and degree properties of individual genes as they relate to the shared neighborhood sizes of their containing WGD pairs.

We calculated Pearson’s correlations for the data. The correlation between shared neighborhood size and the gene length is 0.106, with *P* = 0.0008, and the correlation between shared neighborhood size and the average degree of the two genes is 0.558, with *P* < 2.2*e*−16. These results given the impression of a much stronger correlation between WGD pairs properties and their network divergence than with their sequence divergence. However, one thing to notice is that the shared neighborhood size is highly correlated to the node degrees. If we use shared neighborhood size as a measure of network divergence, then it is possible that all the observations of shared neighborhood size are simply artifacts of degrees in the PPI network. To test this hypothesis, we computed the normalized shared neighborhood size, which is computed as the shared neighborhood size divided by the number of neighbors of either of the genes in the pair. The correlation between normalized shared neighborhood size and the gene length is 0.028, with *P* = 0.3963, the correlation between normalized shared neighborhood size and the copy number is 0.046, with *P* = 0.1612, and the correlation between normalized shared neighborhood size and the average degree of the two genes is −0.099 with *P* = 0.002. In other words, when we normalize the shared neighborhood size, none of the former observed correlations remain significant.

**Figure 4:**
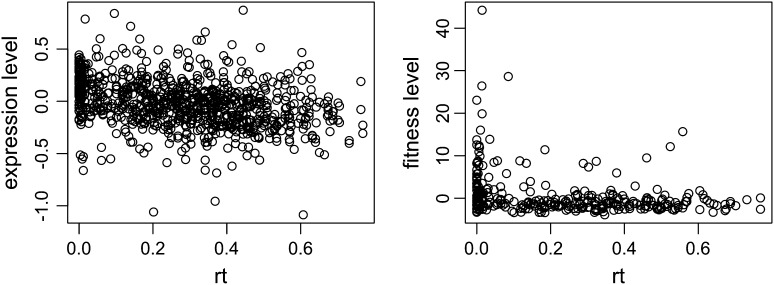
(Left) Expression levels and (right) fitness levels of single genes as a function of the *rt* values of WGD pairs. For a given WGD pair, the expression levels and fitness levels of both genes are plotted individually in the corresponding *rt* value for their containing pair.

Further, we correlated divergence at the sequence level with gene expression and fitness levels. For gene expression levels, we used the data from Spellman *et al.* (1998) and (Tsankov *et al.* (2010). These data are obtained by different groups using different experimental methods, and we apply our analysis to both data sets to validate our results. For gene fitness levels, the data are obtained from Giaever *et al.* (2002), who use five different media under 31 different conditions. We used the normal conditions (condition 18 and 19 in Giaever *et al.*2002) and computed the average fitness values in the five media. Plots of *rt* values *vs.* expression and fitness levels of WGD pairs are given in Figure 4.

The correlation between *rt* and expression levels is −0.3263, with *P* < 2.2*e*−16, indicating that WGD pairs that diverge faster tend to have lower expression levels. The correlation between *rt* and the fitness levels is −0.285, with *P* = 7.16*e*−7, indicating that genes that diverge faster also tends to have lower fitness levels. These strong correlations might have to do with the fates of the duplicated genes, and how redundancy, or lack thereof, created by duplication interplays with fitness effects of the gene pairs. We set out to investigate this by first establishing a connection between WGD pairs evolution and the evolution of their respective interactions in a PPI network, and then learning the fates of duplicated genes from the network topology.

### The rate of PPI evolution as a function of sequence divergence

Recall the definitions of *N_t_*(*g*) and *sh_t_*(*g*_1_, *g*_2_) given previously. Furthermore, we denote by *d_t_*(*g*_1_, *g*_2_) the distance between the two sequences of *g*_1_ and *g*_2_ (in terms of the number of positions they differ at). It is reasonable to assume that *sh*_0_(*g*_1_, *g*_2_) = *N*_0_(*g*_1_) = *N*_0_(*g*_2_) and that *d*_0_(*g*_1_, *g*_2_) = 0. As time progresses, both the sequences of *g*_1_ and *g*_2_ as well as *N_t_*(*g*_1_) and *N_t_*(*g*_2_) begin to diverge, the former due to mutations at the sequence level and the latter due to gain/loss of interactions.

Suppose after some time *T*, we have *d_T_* = *Lp* positions, where *L* = *L*(*g*_1_, *g*_2_) is the length of the aligned portion between the two sequences, and *p* is the proportion of sequence difference at this length. We discard insertions/deletions as the rate of nucleotide substitution is estimated to be orders of magnitude greater than that of insertion and deletion (Saitou 1994). Let us assume that of the *d* differences at time *T*, a proportion of *μ*_ℓ_ result in the loss of new interactions, and a proportion of *μ_a_* result in the gain of new interactions.* That is, *μ*_ℓ_ and *μ_a_* can be thought of as the proportions of sequence substitutions that result in the loss and gain of interactions, respectively. Assuming that *μ*_ℓ_ and *μ_a_* are very small (which is a reasonable assumption), and that in two duplicate genes, all positions in the sequences have identical mutation rates, we obtain

$$sh_{T}\left(g_{1},g_{2}\right)=a{\left(1-\mu_{\ell }\right)}^{d}+d\cdot \mu_{a},$$

where *a* =| *sh*_0_(*g*_1_, *g*_2_)| is the initial number of shared neighbors. The rationale for this equation is as follows. Of *d* mutations, each of the two paralogous genes gains a new edge with rate *μ_a_*, so that the expected number of newly gained edges is *d* ⋅ *μ_a_*. For the shared neighbors, the gain of edges needs to happen for the same neighbor of both *g*_1_ and *g*_2_ or regain a lost edge such that it can contribute to *sh_T_*(*g*_1_, *g*_2_).

Replacing *d* with *Lp* in the aforementioned formula, we obtain *shT*(*g*_1_, *g*_2_) = *a*(1 − *μ*_ℓ_)*^Lp^* + *Lp* ⋅ *μ_a_*. When *μ*_ℓ_ is very small, we have (1 − *μ*_ℓ_)*^L^* ∼1 − *Lμ*_ℓ_. Thus, we obtain

$$sh_{T}\left(g_{1},g_{2}\right)=a{\left(1-L\mu_{\ell }\right)}^{p}+p\cdot L\mu_{a}.$$ (2)

**Figure 5:**
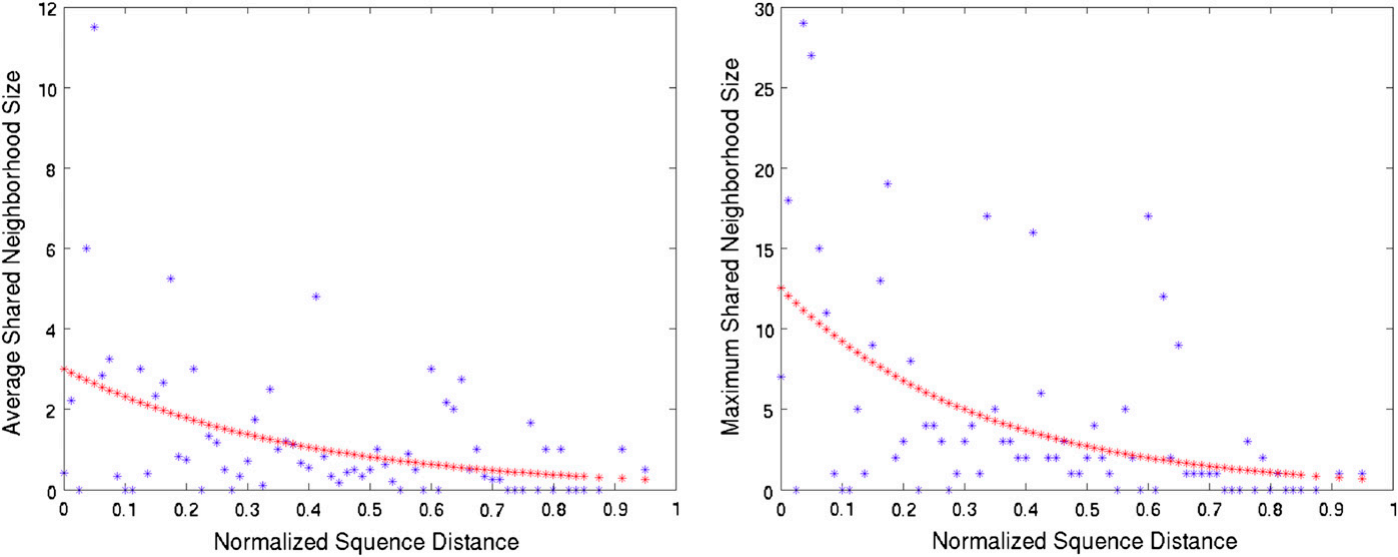
The average (left) and maximum (right) shared neighborhood sizes of WGD pairs as functions of the divergence between the pair’s sequences. The normalized sequence distance is *d*(*g*_1_, *g*_2_)/*L*(*g*_1_, *g*_2_). The red curves are the results of fitting data to Equation (2). Estimated *Lμ*_ℓ_ 0.9261 for the average neighborhood size case and 0.9533 for the maximum neighborhood size case. Estimated *Lμ_a_* is about 0.0001 in both cases.

As we are interested in obtaining estimates of *μ*_ℓ_ and *μ_a_* from WGD pairs, we fit the function in Equation (2) to data obtained from WGD pairs from *S. cerevisiae* that are the result of the WGD event that occurred in yeast approximately 100 million years ago. Because different WGD pairs with the same sequence divergence have different shared neighborhood sizes, we considered both the average and maximum shared neighborhood sizes for given sequence divergence values. Figure 5 shows the results with the function fitting.

In a recent study by Qian *et al.* (2011), the authors experimentally examined 87 potential interactions between *K. waltii* proteins, whose one-to-one orthologs in the related budding yeast *S. cerevisiae* were reported to interact. Their estimate of the evolutionary rate of protein interactions was (2.6 ± 1.6) × 10^−10^ per PPI per year, which is three orders of magnitude lower than the rate of protein sequence evolution measured by the number of amino acid substitutions. In other words, our analysis here provides a similar results based on a different data set. It is interesting to combine these results with the recent findings of (Teichmann and Babu 2004) who showed that about 90% of interactions in transcription regulation networks of *Escherichia coli* and *S. cerevisiae* arose due to gene duplication.

Although our results agree well with the results of Qian *et al.* (2011), the approaches taken are very different. Qian *et al.* (2011). examined PPI divergence after speciation, whereas we examined PPI divergence after WGD. In other words, Qian *et al.* (2011) examined PPIs between interacting pair (*A*, *B*) and their interacting orthologs, or interlogs, (*A*′, *B*′), whereas we examined PPIs between two pairs (*A*, *C*) and (*A*′, *C*) where *A* and *A*′ are paralogs. The fact that all pairs of paralogs we consider are the result of the WGD even in *S. cerevisiae* allows us to use the event as a calibration point and make use of the fact that all pairs have exactly the same age.

It is important to note that the results in Figure 5 are based on data from the DIP database of PPI networks. This database records only high-confidence links and has a relatively high false-negative rate compared with a false positive rate. We repeated the same analysis by using data from the BIOGrid database (with only links that are supported either by low-throughput experiments or by more than a single high-throughput experiment). The trends we obtained are similar to those in Figure 5, with the other difference that the data points and fitted curves are shifted up slightly. The estimated *μ*_ℓ_ and *μ_a_* values were very close to those estimated using the DIP database.

### The fate of WGD gene pairs

**Figure 6:**
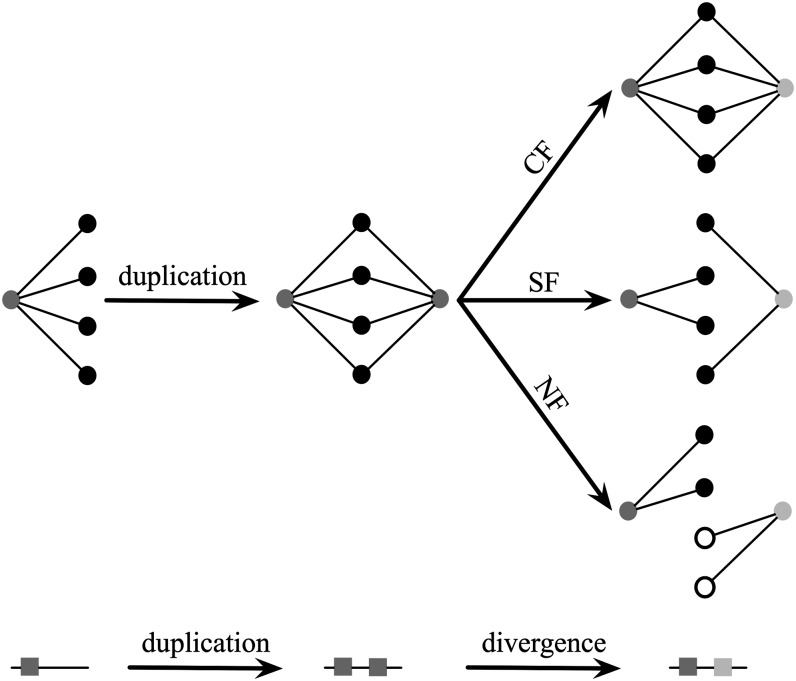
Three fates of a duplicated gene from a network perspective.

After gene duplication, duplicates can have different functional fates, such as maintaining the same function as the ancestral single-copy gene, developing a new function, etc. Given our previous results regarding the use of shared neighborhoods of WGD pairs to estimate the rate of divergence, we here use the neighborhoods of WGD pairs as proxies of their functional fates. For CF, the two genes in a WGD pair maintain exactly the same set of neighbors; in SF, each gene in a WGD pair maintains a subset of original neighbors, whereas the union of their neighbors equals the original set. Finally, in NF, one gene in the WGD pair develops a new set of neighbors while losing all of the duplicated neighbors. According to this strategy, pure conserved functionalization would result in a normalized shared neighborhood size equal to 1, whereas pure subfunctionalization and neofunctionalization would both result in a normalized shared neighborhood size of 0. Figure 6 illustrates these three categories.

**Figure 7:**
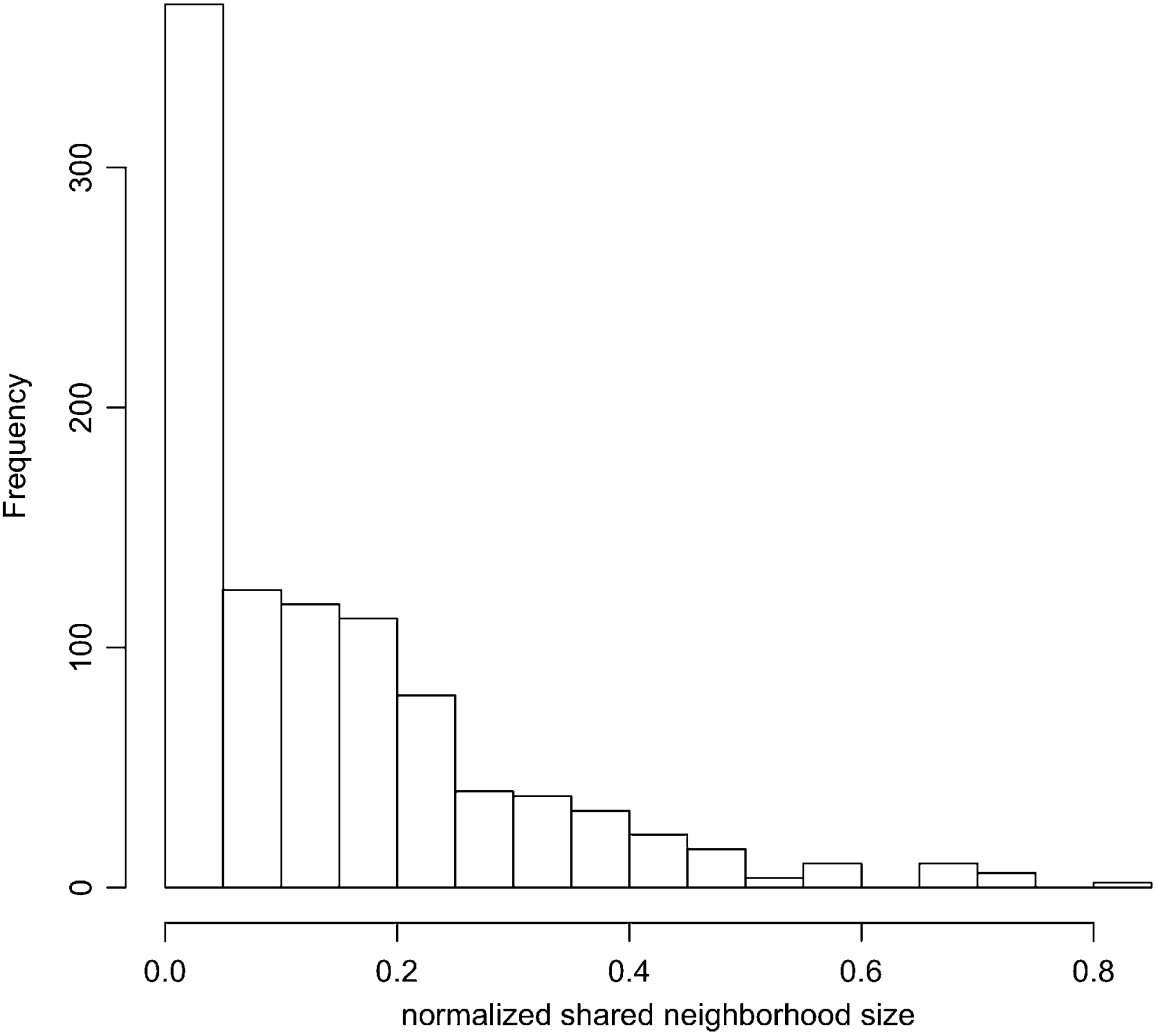
Distribution of normalized shared neighborhood sizes of WGD pairs.

In Figure 7, we show the distribution of normalized shared neighborhood sizes of WGD pairs. As Figure 7 shows, only a very small portion of the WGD pairs actually maintain exactly the same set of neighbors. Approximately 40% of the pairs have totally exclusive neighbors, and most of the gene pairs (60%) share some neighbors while also maintaining some different neighbors. This agrees with the widely known fact that pure SF and NF are rare, and that a large fraction of gene duplicates undergo rapid SF followed by prolonged period of NF referred to as the sub-neo-functionalization model (He and Zhang 2005).

To estimate the actual proportion of gene pairs whose fate is CF, SF, or NF (also, sub-neo-functionalization), we developed an EM algorithm that was inspired by Zeng and Hannenhalli (2013) to estimate the fates from network data (see *Materials and Methods* for full details). Using this algorithm, we estimate that approximately 7−9% of WGD pairs underwent CF, approximately 18−21% WGD pairs underwent NF, and that the rest of WGD pairs (70−75%) underwent SF.

To further explore how these estimated fates correlate with fitness data (as we discussed previously), we categorized gene fitness of WGD pairs by their inferred types. Segre *et al.* (2005) studied the fitness and genetic interactions in yeast on a genome scale and grouped pairs of genes into one of the three categories according to epistasis analysis. Let *w*_1_ and *w*_2_ be the effect on fitness of single-knockout of genes *g*_1_ and *g*_2_, respectively, and let *w*_12_ be the effect on fitness of double-knockout of both *g*_1_ and *g*_2_. Let *e* = *w*_12_ − *w*_1_ ⋅ *w*_2_. By inspecting the *e* values for the different WGD pairs, each pair can be categorized as “no epistasis” (*e* = 0), “aggravating” (*e* < 0), or “buffering” (*e* > 0). We obtained the knockout fitness data from Segre *et al.* (2005) and inspected the epistasis status of the three WGD pair groups (CF, NF, and SF).

**Figure 8:**
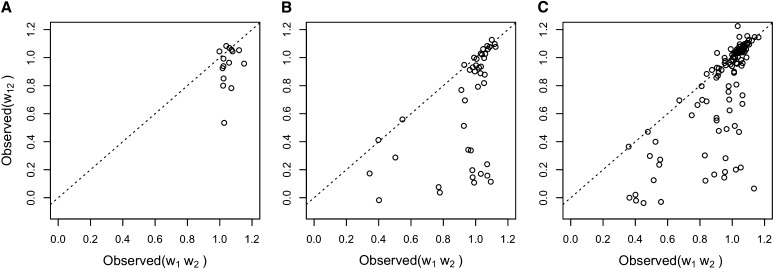
Fitness effects of double-knockouts of WGD pairs in the groups (A) CF, (B) NF, and (C) SF. The dashed line corresponds to no epistasis, whereas the regions above and below the line correspond to buffering and aggravating epistasis, respectively.

For all 550 WGD pairs, only 182 pairs have both PPI data for inferring duplication type based on our methodology and data from epistasis analysis. The values of *w*_12_ and *w*_1_ ⋅ *w*_2_ for WGD pairs in the three groups are shown in Figure 8.

For the SF group, 2 pairs have no epistasis, 45 pairs are buffering, and 77 pairs are aggravating. For the NF group, 0 pairs have no epistasis, 11 pairs are buffering, and 32 pairs are aggravating. For the CF group, 0 pairs have no epistasis, 3 pairs are buffering, and 12 pairs are aggravating. Overall, WGD gene pairs tend to have more of a buffering epistatic effect, and the trend is more obvious when the duplication pairs evolve with CF.

Dean *et al.* (2008) pointed out that most duplicated genes are functionally redundant. For essential reactions, only 0.2% show negative epistasis. For nonessential reactions, 4% show negative epistasis. Our results show that WGD pairs have high proportion with negative epistasis, which means WGD genes are highly redundant. Also, the SF group has the lowest ratio of aggravating pairs while CF group has the highest ratio of aggravating pairs. This indicates that CF group is most functional redundant among the three groups, which makes sense given the conserved functionality. Furthermore, these result demonstrate the utility of using network structure for determining the evolutionary fate of gene duplicates.

## Discussion

In this work, we took a network perspective on the evolution of WGD pairs and investigated WGD pairs in yeast with respect to the yeast’s PPI network. The calibrated time of all gene pairs in this data set makes it an ideal data set for understanding evolution of gene duplications. We correlated divergence of WGD duplicates at the sequence and network level. Further, we demonstrated strong correlations between WGD pair divergence and fitness. Finally, using the neighbors of WGD pairs as proxies for the functions of genes in these pairs, we developed a method to infer the evolutionary fate of WGD pairs and then correlated the categories of WGD pairs with different fates with fitness effects. Our results indicate that network connectivities can provide a powerful tool to investigate and understand the evolution of gene duplicates.

Notice that the estimated *μ_a_* is much smaller than *μ*_ℓ_, which means that during evolution, the chance to add an edge for one or both gene in the duplicated pair is about three orders of magnitude smaller than deleting an edge. This agrees with the hypothesized DMC (duplication-mutation with complementarity) model (Middendorf *et al.* 2005) of network evolution regarding gene divergence after duplication. In other words, these types of analyses can help inform whether commonly used models of network evolution are plausible, as well as derive new ones.

It is important to point out that network data does not come without error. Indeed, network data are very erroneous when compared, for example, with sequence data. Although we conducted our analyses independently with two sources of data (DIP and BioGrid), and despite the good agreement between the two, we still expect inaccuracies of network data to be present and affect the results. As technologies for deriving interaction data continue to improve, it would be interesting to apply these methods to more accurate network data.

Another factor that could affect our results is gene conversion, because interlocus conversion events that occurred after WGD significantly affect the estimated sequence divergence and, consequently, the correlations between sequence and network divergence. It is estimated that only approximately 10% gene pairs underwent gene conversion, and it would be interesting to investigate how gene conversion comes into play between sequence and network divergence.

**Figure 9:**
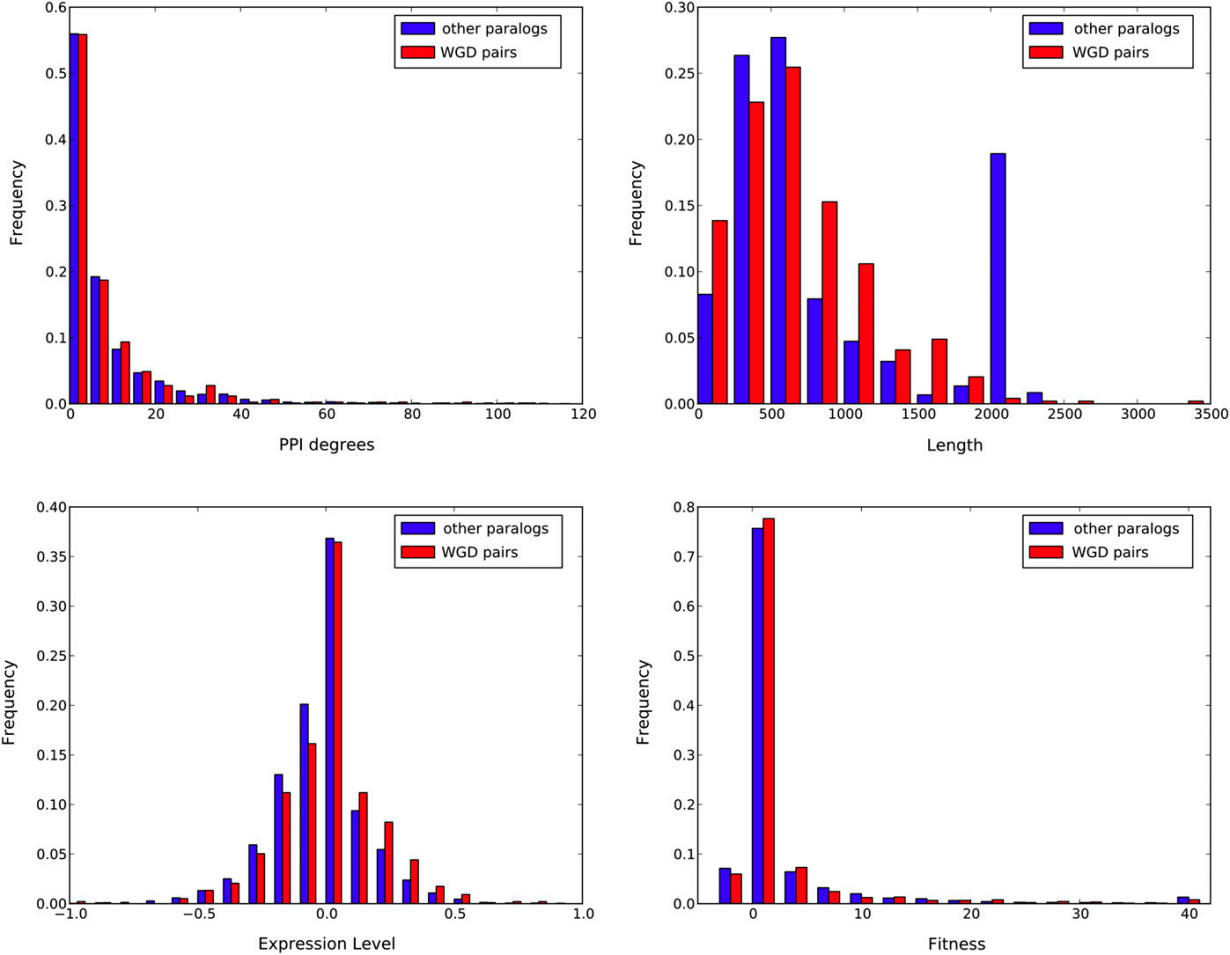
The PPI degree, gene length, gene expression level, and fitness level of WGD pairs and non-WGD pairs are shown.

Finally, a question naturally arises as to whether WGD pairs are a good representative of gene duplicate pairs in general. To investigate this question, we inspected four properties of WGD and non-WGD pairs: PPI degrees, lengths of gene sequences, expression levels, and fitness values. The results are shown in Figure 9.

The figure clearly shows that with the exception of gene lengths, WGD and non-WGD pairs agree in terms of other properties. These results indicate that WGD pairs provide a good sample of gene duplicates in general. Given the knowledge about their duplication time, they are the ideal candidates of gene duplications to shed light on network evolution, and to translate network-based information from WGD pairs to general duplicate pairs. These results further highlight the significance of our findings on modeling network evolution and developing model-based methods for ancestral network reconstruction (Navlakha and Kingsford 2011; Zhu and Nakhleh 2012).

*Here, we are assuming that gain and loss of interactions are governed mainly by substitutions at the sequence level.
